# Data on synthesis and thermo-mechanical properties of stimuli-responsive rubber materials bearing pendant anthracene groups

**DOI:** 10.1016/j.dib.2016.09.023

**Published:** 2016-09-22

**Authors:** Jakob Manhart, Santhosh Ayalur-Karunakaran, Simone Radl, Andreas Oesterreicher, Andreas Moser, Christian Ganser, Christian Teichert, Gerald Pinter, Wolfgang Kern, Thomas Griesser, Sandra Schlögl

**Affiliations:** a Polymer Competence Center Leoben GmbH, Roseggerstraße 12, A-8700 Leoben, Austria; b Christian Doppler Laboratory for Functional and Polymer Based Ink-Jet Inks, Otto Glöckel-Straße 2, A-8700 Leoben, Austria; c Chair of Materials Science and Testing of Plastics, University of Leoben, Otto Glöckel-Straße 2, A-8700 Leoben, Austria; d Institute of Physics, University of Leoben, Franz Josef – Straße 18, A-8700 Leoben, Austria; e Christian Doppler Laboratory for Fiber Swelling and Paper Performance, Graz University of Technology, Inffeldgasse 23, 8010 Graz, Austria; f Chair of Chemistry of Polymeric Materials, University of Leoben, Otto Glöckel-Straße 2, A-8700 Leoben, Austria

**Keywords:** Photoreversible networks, Reaction kinetics, Thermo-mechanical properties

## Abstract

The photo-reversible [4πs+4πs] cycloaddition reaction of pendant anthracene moieties represents a convenient strategy to impart wavelength dependent properties into hydrogenated carboxylated nitrile butadiene rubber (HXNBR) networks. The present article provides the ^1^H NMR data on the reaction kinetics of the side chain functionalization of HXNBR. 2-(Anthracene-9-yl)oxirane with reactive epoxy groups is covalently attached to the polymer side chain of HXNBR *via* ring opening reaction between the epoxy and the carboxylic groups. Along with the identification, ^1^H NMR data on the quantification of the attached functional groups are shown in dependence on reaction time and concentration of 2-(anthracene-9-yl)oxirane. Changes in the modification yield are reflected in the mechanical properties and DMA data of photo-responsive elastomers are illustrated in dependence on the number of attached anthracene groups. DMA curves over repeated cycles of UV induced crosslinking (*λ*>300 nm) and UV induced cleavage (*λ*=254 nm) are further depicted, demonstrating the photo-reversibility of the thermo-mechanical properties. Interpretation and discussion of the data are provided in “Design and application of photo-reversible elastomer networks by using the [4πs+4πs] cycloaddition reaction of pendant anthracene groups” (Manhart et al., 2016) [1].

**Specifications Table**

**Table:** 

Subject area	*Chemistry*
More specific subject area	*Polymer Photochemistry*
Type of data	*Table, graphs*
How data was acquired	^*1*^*H NMR spectra were recorded with a Varian 400-NMR operating at 399.66* *MHz and DMA measurements were performed with a SDTA861E DMA analyzer from Mettler-Toledo.*
Data format	*Analyzed*
Experimental factors	^*1*^*H NMR spectra were acquired by using a relaxation delay of 10s and 45*° *pulse. CDCl*_*3*_*was used as solvent and spectra were referenced to Si(CH*_*3*_*)*_*4*_*as internal standard. For DMA measurements, an amplitude of 20* *µm and a measurement frequency of 1* *Hz were used. The test specimen were heated from −60* *°C to +40* *°C with a heating rate of 2* *K/min.*
Experimental features	*Functional rubber materials were synthetized and*^*1*^*H NMR experiments were performed to verify and quantify the attachment of 2-(anthracene-9-yl)oxirane. Loss factor and storage modulus of photo-reversible rubber materials are provided in dependence on the modification yield and UV exposure dose.*
Data source location	*Leoben, Austria*
Data accessibility	*Data are provided with this article*

**Value of the data**

• ^1^H NMR data enables an easy assigning of the proton peaks, which is relevant to determine the modification yield of side chain modified rubber and polymer materials.
• Comparison of the reaction kinetics with other epoxy based systems can help to understand the influence of key parameters on the ring-opening reaction between epoxy moieties and carboxylic acid groups.
• DMA data allows a deeper understanding of the role of bulky aromatic side chains on the structural-property relationship of elastomer materials.

## 1 Data

^1^H NMR data on the verification and quantification of HXNBR materials with pendant anthracene groups are provided. DMA data on side chain functionalized HXNBR materials are shown in dependence on the modification yield.

## 2 Experimental design, materials and methods

### 2.1 ^1^H NMR experiments of side chain functionalized HXNBR

The synthesis procedure is provided in Ref. [1]. For the characterization of the reaction kinetics, small amounts of the reaction solution were taken out at selected reaction times of one batch that was stirred at room temperature for 72 h. The reaction solution was immediately precipitated to stop the ongoing reaction and subsequently two purification steps were carried out in which the samples were dissolved in chloroform again and precipitated in cold methanol. After removing the supernatant from the precipitate, the latter was dried at room temperature under vacuum and analyzed regarding its modification yield.

For the quantification of the modification yield, ^1^H NMR spectra were referenced to a residual chloroform signal of 7.27 ppm (corresponding to Si(CH_3_)_4_ with a shift of 0.0 ppm) after Fourier transformation. In order to quantify the anthracene signal, the isolated peak at 4.05 ppm was integrated including its complete shoulders on both sides after an appropriate baseline subtraction. For reference purposes, the signal from 2.95 to 0.2 ppm was integrated as it originates from the polymer chain. The modification yield was then calculated according to Eq. (1), wherein the average number of protons per repeating unit was calculated to be 6.033.

(1) $$\mathrm{modification}\;\mathrm{yield}=\frac{\mathrm{single}\;\mathrm{proto}n^{\prime }s\;\mathrm{signal}\;\mathrm{of}\;\mathrm{attached}\;\mathrm{anthracene}}{\left(\frac{\mathrm{signal}\;\mathrm{of}\;\mathrm{polymer}\;\mathrm{chain}}{\mathrm{average}\;\mathrm{number}\;\mathrm{of}\;\mathrm{protons}\;\mathrm{per}\;\mathrm{repeating}\;\mathrm{unit}}\right)}$$

**Fig. 1:**
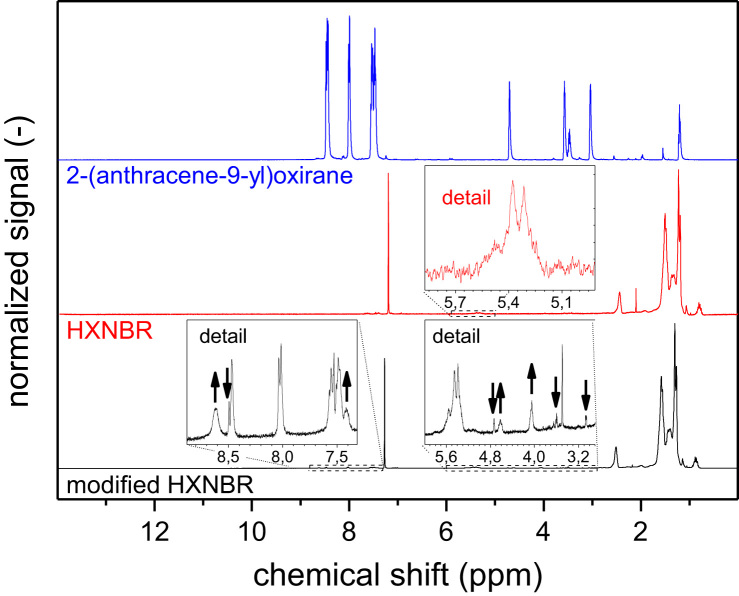
^1^H NMR spectra of 2-(anthracene-9-yl)oxirane, HXNBR and their reaction product (arrows indicate peaks that emerged and decreased upon side chain functionalization).

Fig. 1 provides the ^1^H NMR spectra of HXNBR prior to and after side chain modification with 2-(anthracene-9-yl)oxirane together with the ^1^H NMR spectrum of 2-(anthracene-9-yl)oxirane.

**Fig. 2:**
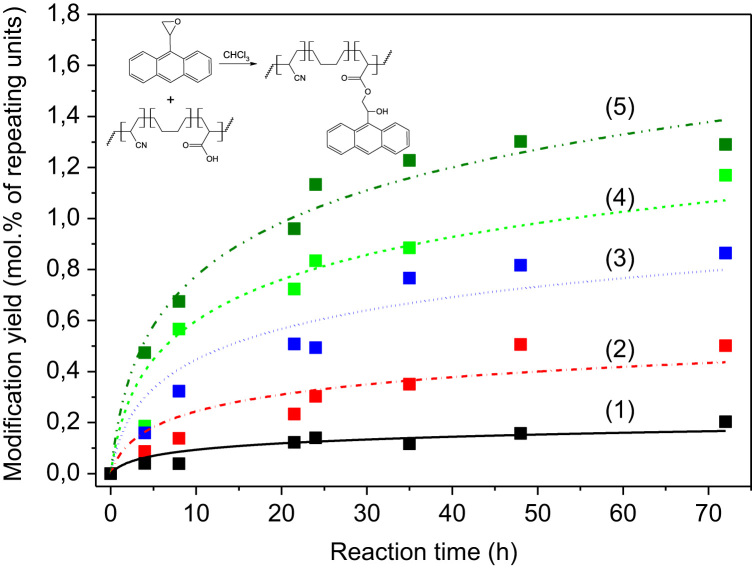
Modification yield of photo-responsive rubber materials *versus* reaction time in dependence on the 2-(anthracene-9-yl)oxirane concentration; (1) 10, (2) 30, (3) 60, (4) 100 and (5) 150 phr (parts per hundred rubber). (The line is a guide to the eye).

Fig. 2 shows the reaction kinetics of the side chain modification of HXNBR, which was carried out at room temperature. The number of attached anthracene groups is plotted against the reaction time. The influence of the concentration of 2-(anthracene-9-yl)oxirane in the reaction mixture on the modification yield is displayed.

### 2.2 DMA experiments of side chain functionalized HXNBR

Sample preparation and DMA experiments are detailed in Ref. [1]. Table 1 illustrates the storage modulus at 23 °C of side chain modified HXNBR materials in dependence on the modification yield that ranges from 0 to 1.30 mol%.

**Fig. 3:**
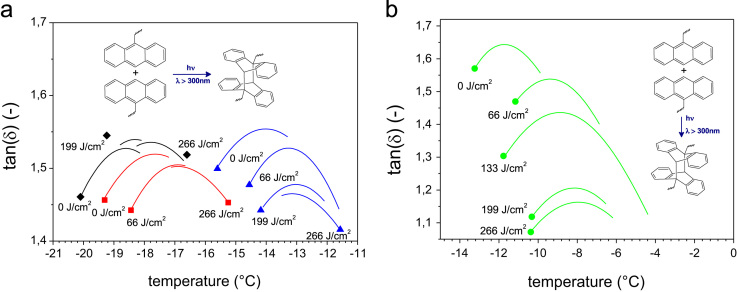
DMA curves of photo-responsive rubber materials upon prolonged UV exposure (*λ*>300 nm); (a) (♦) rubber-**0**, (■) rubber-**1**, (▲) rubber-**2** and (b) (•) rubber-**3**.

**Fig. 4:**
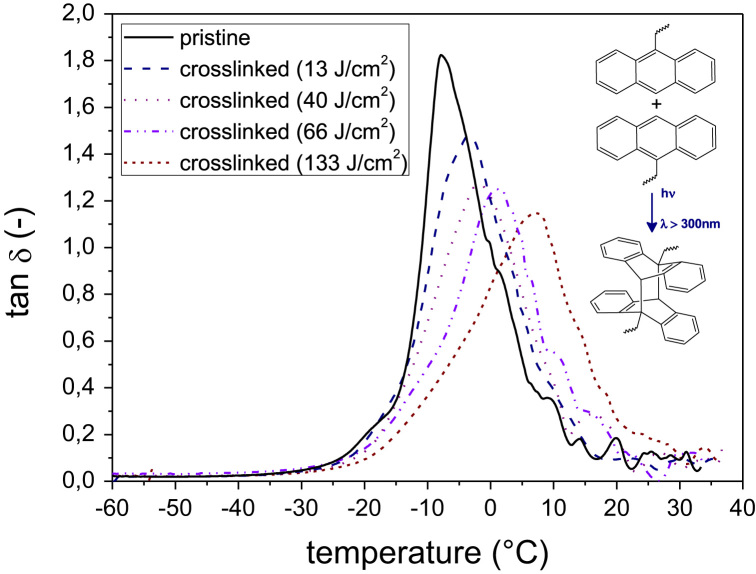
DMA curves of rubber-**4** upon prolonged UV exposure (*λ*>300 nm) over a temperature range from −60 to +40 °C. Details of the DMA curves in a narrow temperature range are also given in Ref. [1].

Fig. 3a and b provide the DMA curves of photo-responsive rubber materials with different modification yields, which have been photochemically crosslinked (*λ*>300 nm) at different exposure doses. In addition, Fig. 4 details the DMA curves of rubber-**4** in a broad temperature range over prolonged UV exposure (*λ*>300 nm).

**Fig. 5:**
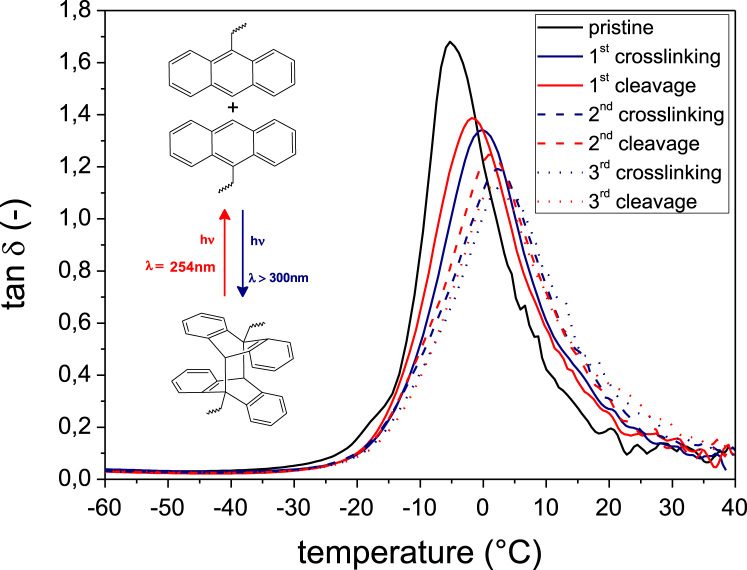
DMA curves of rubber-**4** over repeated cycles of UV induced crosslinking with 46 J/cm^2^ (*λ*>300 nm, N_2_) and UV induced cleavage with 9.72 J/cm^2^ (*λ*=254 nm, N_2_) over a temperature range from −60 to +40 °C. Details of the DMA curves in a narrow temperature range are also given in Ref. [1].

In Fig. 5 the DMA curves of rubber-**4** are provided in a broad temperature range over three cycles of photochemical crosslinking and subsequent photochemical cleavage upon deep UV exposure.

## Figures and Tables

**Table 1:** Storage modulus (*E*′) of photo-responsive rubber materials after UV induced crosslinking.

Sample	Modification yield^a^/mol.%	*E*′ at 23 °C after UV exposure (260 J/cm^2^)^b^/MPa
Rubber-**0**	0	2.7
Rubber-**1**	0.19	2.7
Rubber-**2**	0.49	2.9
Rubber-**3**	0.86	3.1
Rubber-**4**	1.30	3.3

a Determined by ^1^H NMR experiments.

b Determined by DMA measurements.
