# Supplementary material for: Data on synthesis and thermo-mechanical properties of stimuli-responsive rubber materials bearing pendant anthracene groups
Source: Data Brief. 2016 Sep 22;9:524–9. doi: 10.1016/j.dib.2016.09.023 (PMC5054239; doi:10.1016/j.dib.2016.09.023)
Supplement: Supplementary file 1 — Supplementary material [file mmc1.pdf]

Polymer Competence Center Leoben

Dr. Sandra Schlögl
Polymer Competence Center
Leoben GmbH
Roseggerstrasse 12
A – 8700 Leoben, Austria

phone ++43 3842 402 - 2354
fax ++43 3842 402 - 2352
sandra.schloegl@pccl.at

Leoben, 10th Sept., 2016

AUTHOR DECLARATION

Title: **"Data on synthesis and thermo-mechanical properties of stimuli-responsive rubber materials bearing pendant anthracene groups"**

Corresponding and submitting author: *Sandra Schlögl*

Co-authors: *Jakob Manhart, Santhosh Ayalur-Karunakaran, Simone Radl, Andreas Oesterreicher, Andreas Moser, Christian Ganser, Christian Teichert, Gerald Pinter, Wolfgang Kern and Thomas Griesser*

The authors wish to confirm that there are no known conflicts of interest associated with this publication and there has been no significant financial support for this work that could have influenced its outcome.

The authors confirm that the manuscript has been read and approved by all named authors and that there are no other persons who satisfied the criteria for authorship but are not listed. The authors further confirm that the order of authors listed in the manuscript has been approved by all of them.

The authors confirm that all authors have given due consideration to the protection of intellectual property associated with this work and that there are no impediments to publication, including the timing of publication, with respect to intellectual property. In so doing the authors confirm that the authors have followed the regulations of their institutions concerning intellectual property.

The authors understand that the Corresponding Author is the sole contact for the Editorial process (including Editorial Manager and direct communications with the office). She is responsible for communicating with the other authors about progress, submissions of revisions and final approval of proofs.

Signed by the corresponding author on behalf of all authors.

Leoben, 10th September, 2016

Sandra Schlögl

Dr. Sandra Schlögl
